# Randomized Controlled Trial Designs for Operations Research in Low-Income Countries: Reality or Delusion?

**DOI:** 10.3389/fpubh.2013.00014

**Published:** 2013-05-06

**Authors:** Setor K. Kunutsor, John Walley

**Affiliations:** ^1^Department of Public Health and Primary Care, University of CambridgeCambridge, UK; ^2^Nuffield Centre for International Health and Development, Institute of Health Sciences, Leeds UniversityLeeds, UK

Operational research is an approach that is gaining ground in low-income countries. It is used to evaluate the effectiveness and feasibility of innovative interventions and helps to influence policy, practice, and improve program performance (1). In low-income settings where the burden of disease is high and resources are very constrained, efficiency of health systems can be improved by conducting research that is embedded within national health program and makes use of existing resources. Elements of operational research include generating research questions based on service delivery needs and constraints identified within national programs, designing and planning the research study within the context of the program, and implementation of the interventions by the program with the research team facilitating the process (1, 2). There is a large body of evidence on the importance of operational research in low-income settings and its impact on policy and practice (1, 3). Various donor organizations and governments have stressed the need for national health programs to include operational research studies as routine activities within their programs (4, 5). But this is a key issue for low-income countries, with the current global economic turndown and the already scarce resources in these settings. It is however hoped that with the commitment by some donor agencies to fund operational research (6) and the increase in the proportion of Global Fund grants for operational research (7), its integration into programs in low-income settings will become a reality.

Operational research studies have commonly used cross-sectional, case-control, or cohort designs, with Zachariah and colleagues (1) being of the view that operational research should not include randomized controlled trial (RCT) designs. We concur with Horstick and colleagues (8) on the importance of including a broader spectrum of research designs such as randomized trials in this kind of research. Studies conducted in Uganda and elsewhere, have shown that RCTs pragmatically designed can be used to answer operations research questions. Our RCT conducted in Uganda (9) which assessed the effectiveness of the treatment supporter strategy in improving adherence to antiretroviral therapy, was conducted with minimum additional inputs with the implementation of the adherence intervention under routine care conditions. Chang and colleagues (10) during their cluster randomized trial to assess the effect of community-based peer health workers on AIDS care of adults in Uganda, made use of ongoing program care conditions despite their complex interventions.

Randomized controlled trials are powerful tools and considered to be the best of research designs in evaluating the efficacy of interventions (11). Their main strength lies in the randomization procedure which has the potential to reduce bias. RCTs are however not without their drawbacks especially in the context of low-income settings. They may not be feasible in situations of financial constraints or where there are high drop-out rates or low compliance among study participants. Also due to ethical dilemmas and practical constraints, some important aspects of healthcare cannot be subjected to a RCT design (12). Zachariah and colleagues (1) argue against their inclusion in operational research designs because they assess the effectiveness of interventions in tightly controlled environments in selected populations as opposed to the routine program settings of operational research. However, the experience in Uganda and other low-income countries, shows that operational research can use RCT designs and be conducted within ongoing program conditions, provided it is considered ethically acceptable, the design of the intervention and tools is for the routine setting, and it is based on existing resources and data systems such as treatment registers. Key elements of the RCT conducted in Uganda included generation of the research questions based on challenges identified within the National AIDS Control Program, involvement and engagement of program staff right from the outset, use of data being collected under routine care conditions, and making use of existing usual program staff and other resources. Developmental activity included modification of educational materials and data-collection tools already available and training of health workers to implement interventions. The implementation can be done, as in the Uganda example, by the health service staff. In addition, a competent research officer was employed to work alongside the program staff to facilitate the whole study. Implementing the intervention was not without challenges such as divergent priorities of busy program staff and some resource constraints. It was also not possible to eliminate ascertainment and observation bias as blinding the interventions to the patients and program staff would be unethical and not feasible in that particular situation. Overall, financial constraints were not an issue during the conduct of the study. Furthermore, in operational research studies where the intervention is at the district level or several facilities are involved, a cluster RCT is preferred to the individually RCT design as demonstrated by Chang and colleagues (10). In Cluster RCT designs, participants are not allocated to the interventions individually, but as a group (13). Cluster RCT designs are also advantageous when there is too great a risk of “contamination” between the intervention and control arms (e.g., usual care) (14). Drawbacks to the cluster RCT designs however, are their complexity in design and analysis, high costs may be involved, and the need for an increased sample size to obtain sufficient power (15). Further discussion is warranted on this topic. We have recently commissioned three operational research projects using cluster RCT designs in Pakistan and China within our health service delivery program consortium (16).

Randomized controlled trial designs are in principle simple and yet are the most powerful tools of research and are traditionally the “gold standard” for judging the benefit of an intervention. We feel RCTs are feasible and appropriate in operational research settings. When incorporating RCT designs in operations research, ethical issues such as acting in the best interests of the participants/patients and equipoise (existing uncertainty about the effect of the intervention to be evaluated (12)) should be addressed. We recommend that operational research groups consider including RCTs where appropriate in their research designs.
